# Synchronized moulting behaviour in trilobites from the Cambrian Series 2 of South China

**DOI:** 10.1038/s41598-020-70883-5

**Published:** 2020-08-24

**Authors:** Alejandro Corrales-García, Jorge Esteve, Yuanlong Zhao, Xinglian Yang

**Affiliations:** 1 Department of Geosciences, Universidad de Los Andes, 111711 Bogotá D.C., Colombia; 2 College of Resources and Environmental Engineering College, Guizhou University, Guiyang, 550025 China; 3 Key Laboratory of Geological Resources and Environment, Ministry of Education, Guizhou University, Guiyang, 550025 China

**Keywords:** Palaeontology, Palaeoecology

## Abstract

The study of moulting behaviour in the fossil record is relatively well known in arthropods and this is especially true for trilobites. Nevertheless, while studies focusing on the style of moulting in social and semi-social groups of modern animals (e.g. arthropods) are common, very few works investigate moulting adaptations in deep time. Here we report a trilobite assemblage from the Cambrian Series 2 “Tsinghsutung” Formation of South China. Around 850 specimens were used for this study from three different levels across one section near Balang (SE Guizhou Province, South China). These levels preserve numerous trilobite clusters in some cases containing around 400 individual specimens. Up to four species have been found in these clusters, but two species are more common. Trilobite clusters bear a high percentage of disarticulated specimens that we interpret as moults. Additionally, measurements of bioclast orientation and the dorsoventral attitude suggests very quiet water conditions followed by rapid burial events, prior to scavenger disturbance. Together, this indicates that the fossil assemblages were a result of a biological phenomenon rather than mechanical processes, allowing us to interpret the position of the fossil parts as different moulting configurations. Since the trilobite assemblage seems to be in situ, the large number of exuviae suggests a local place of migration. This was triggered by the need for group protection while moulting, which is suggestive of gregarious behaviour, possibly synchronized. These trilobites from the Cambrian Epoch 2, Age 4 constitute one of the earliest known gregarious community of trilobites and has important implications for understanding the ecology of this group during their emergence in the Cambrian.

## Introduction

Arthropods (i.e. insects, spiders, crustaceans, myriapods and others), are the most successful Phanerozoic animals. This group of segmented jointed-limbed animals is characterized by the possession of a hard cuticle that is episodically moulted. Moulting allows growth but also occasionally repairs injures of the old exoskeleton^1,2^. However, moulting represents a risk, as for a short period of time after moulting, the new exoskeleton is too soft for protection against predators and conspecifics^3–6^. Consequently, arthropods have developed different morphological adaptations and behaviours to overcome this problem^3^. Morphological adaptations such as cuticle thickening or spines appear early in the fossil record and are likely a response to predation^7^. Behavioural adaptations, on the other hand, represent a more complicated form of protection and require a high degree of social organization^5,8–12^. One of these adaptations is the synchronized moulting seen in modern social and subsocial groups of arthropods such as spiders, shrimps, prawns, crabs and springtails^5,8–11^ and its presence is being increasingly observed in the fossil record (e.g. trilobites, eurypterids, megacheirans and other crusaceomorphs, see^12–18^).

Trilobites, as part of the Arthropoda clade, were no exception to this behaviour. Together with eurypterids and crustaceans, trilobites contribute the majority of the data available regarding moults in the fossil record^6^. However, research has predominantly been focused on the description of the moulting mechanics rather than the evolutionary and ecological importance of moulting in extinct species^2,6^. Synchronized moulting behaviour in trilobites has been previously suggested for assemblages of carcasses and moults of *Balcoracania dailyi* Pocock, 1970^19^, *Homotelus bromidensis* (Esker, 1964)^20^ (see^14,16^) and the linear clusters of *Ampyx priscus* Thoral, 1935^21^ in Vannier et al*.*^18^. Like in other groups of arthropods, synchronized moulting in trilobites may also be due to a variety of abiotic factors, such as temperature, tides or photoperiod and lunar cycles, or due to biotic factors such as predators, age, reproductive status or feeding (see^8,17,22^). In this study we report and discuss the early evidence for gregarious moulting of two oryctocephalid species from the ‘Tsinghsutung’ Formation, Cambrian Series 2 (Stage 4) of Balang, South China.

## Results

### Trilobite preservation

**Figure 1:**
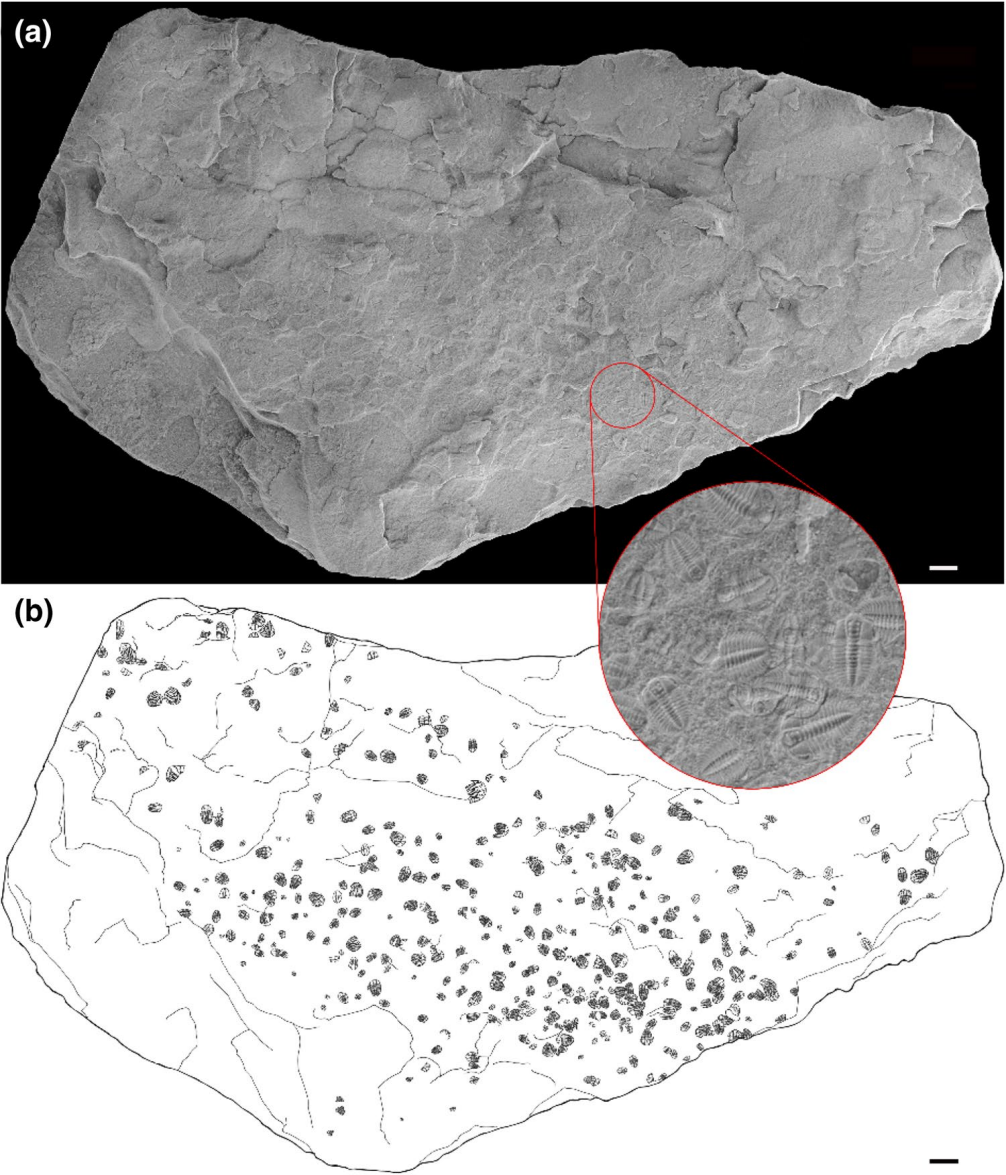
Slab from Q52 showing the biggest trilobite cluster **(a)** picture of the sample; **(b)** drawing showing the trilobite distribution (n = 854). Scale bar = 1 cm.

Specimens in this study come from three levels in the ‘Tsinghsutung’ Formation, Q51, Q52 and Q53, and are preserved in the majority of cases as complete individuals (i.e. articulated), although some sclerites are absent in a few cases (see moulting configurations and discussion below) (Fig. 1, see Fig. 1S supplementary material). Trilobites from these levels are typically preserved through replacement by the mineral illite. The illite degrades into iron oxides, and after dissolution has the ability to produce good quality internal and external moulds^23,24^.

### Bioclast abundance, composition and articulation

**Figure 2:**
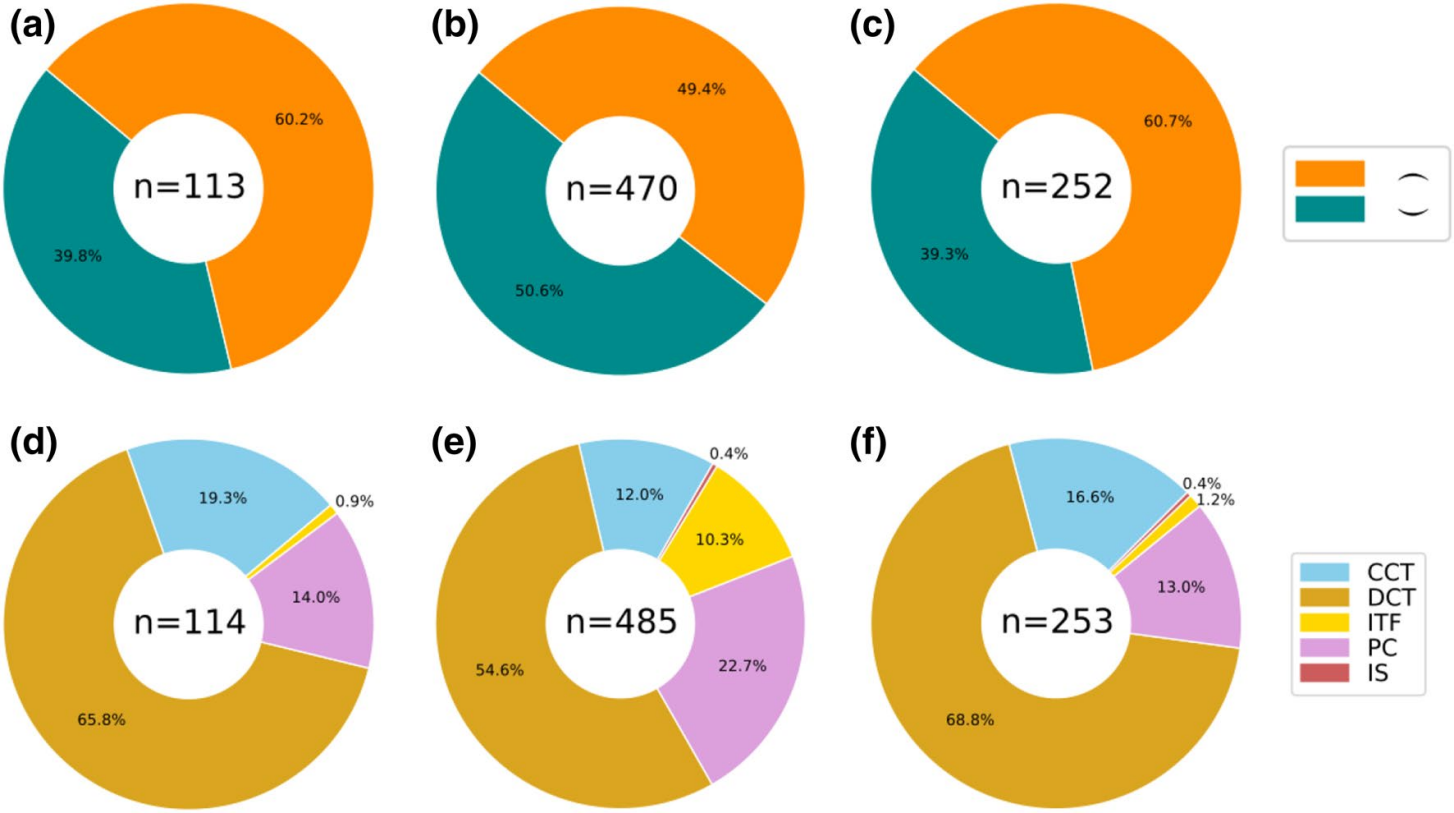
**(a–c)** Bioclast dorso-ventral attitude; **(a)** Q51; **(b)** Q52; **(c)** Q53. **(d–f)** Bioclast composition; **(d)** Q51; **(e)** Q52; **(f)** Q53. *CCT* complete cephalotrunk, *DCT* disarticulated cephalotrunk, *ITF* isolated thoracic fragment, *PC* part of cephalon, *IS* isolated sclerite. Plotted using Matplotlib library^65^.

Bioclasts in the slabs are densely packed aggregates of semi-articulated specimens, sometimes concentrated in a specific region of the slab and are occasionally overlapping (Fig. 1). Clusters consist of 4 to 407 individuals and, although isolated specimens were also found, they were unusual in these three beds. A total of 857 individuals were included in this analysis (114 in Q51, 488 in Q52 and 255 in Q53). The clusters are mostly formed by complete or nearly complete individuals with a very high percentage of articulation, the majority being complete specimens with absent ventral cephalic structures (Fig. 2). The absence of ventral cephalic structures indicates that the majority of these specimens are moults. The abundance of bioclasts also varies within slabs with the density of individuals fluctuating across the slabs (see Fig. 1), but those were never compacted enough to suggest a hash-surface sensu Webster et al*.*^23^. Here we divided the bioclast composition into five different categories (Fig. 2) predominantly based on the definitions outlined by Webster et al*.*^23^: complete cephalon-trunk (CCT), disarticulated cephalon-trunk (DCT), isolated part of cephalon (IPC), isolate trunk fragment (ITF) and isolated sclerites (IS). Even though isolated sclerites of trilobites are common in the fossil record, especially in Lagerstätten^25^, this study shows the opposite trend, as the most common kind of bioclasts are highly articulated specimens (Fig. 2), suggesting low current flows and rapid burial deposition. Samples of DCT show the highest percentage of individuals (ca. 60.3%) (Predominantly only lacking the ventral cephalic structures, ca. 82,33%), followed by CCT (ca. 18.7%), with 79% very articulated individuals versus 20.6% isolated fragments of trunk (ITF) (ca. 14.3%) and cephalon (IPC) (ca. 6.3%); and finally a very small component of isolated trunk sclerites (IS) (ca. 0.4%). These proportions of trilobite elements suggest moulting configurations rather than disarticulation due to the environment (see moulting configuration).

### Taxonomic composition

**Figure 3:**
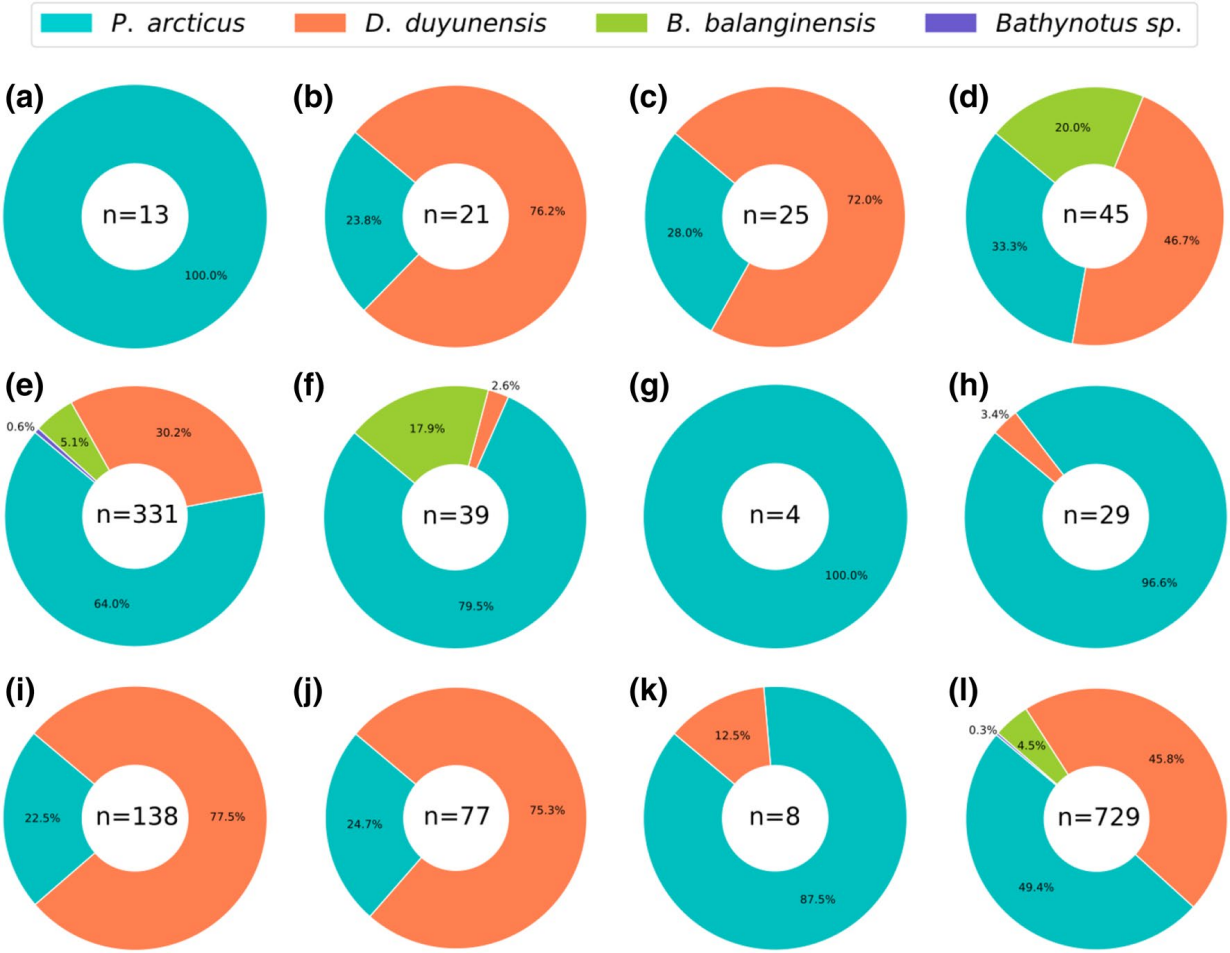
Percentage of taxonomic composition for each one of the slabs **(a)** Q51-1207, **(b)** Q51-1213, **(c)** Q51-1216, **(d)** Q51-12188, **(e)** Q52-348, **(f)** Q52-2391, **(g)** Q52-4675, **(h)** Q52-674, **(i)** Q53-15, **(j)** Q53-461, **(k)** Q53-933-8941, and **(l)** total. **(a–d)** Q51; **(e–h)** Q52; **(i–k)** Q53; and **(l)** total. Plotted using Matplotlib library^65^.

Four species were found in these three beds: two oryctocephalids (*Protoryctocephalus arcticus* Geyer & Peel, 2011^26^ and *Duyunaspis duyunensis* Zhang & Qian in Zhou et al*.*, 1977^27^), one burlingiid (*Burlingia balangensis* Yuan & Esteve, 2015^28^) and an isolated poorly preserved cranidia of cf. *Bathynotus* in the Q52 (Fig. 3). Although *P. arcticus* and *D. duyunensis* seem to be codominant, in each single plate the dominance of one species is clearly showing segregation (Fig. 3a–k). *P. arcticus* was the most abundant in the largest slabs belonging to Q52 (Figs. 1, 3), however, *D. duyunensis* was the most commonly found species in most slabs from Q51 and Q53. *B. balangensis* was rarely observed and completely absent in Q53 (Fig. 3).

### Bioclast size distribution and ontogeny

**Figure 4:**
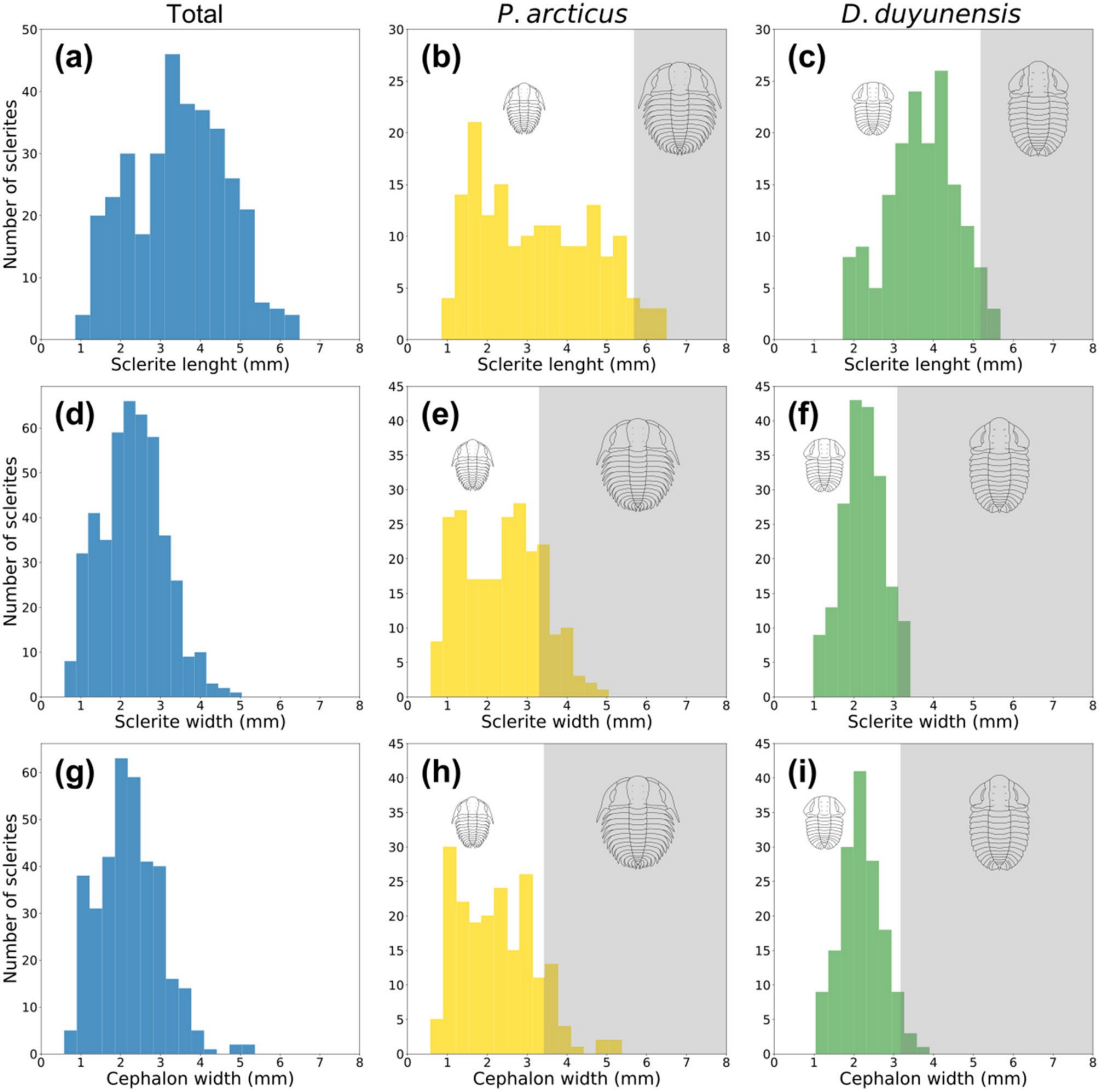
Size distribution of complete sclerites. **(a–c)** Sclerite length; **(e–g)** sclerite width; **(h–j)** cephalic width. Analysis for all individuals in **(a)**, **(e)** and **(h)** *P. arcticus* in **(b)**, **(f)** and **(i)** and *D. duyunensis* in **(c)**, **(g)** and **(j)**. Gray area indicates holaspid individuals. Plotted using Matplotlib library^65^.

**Figure 5:**
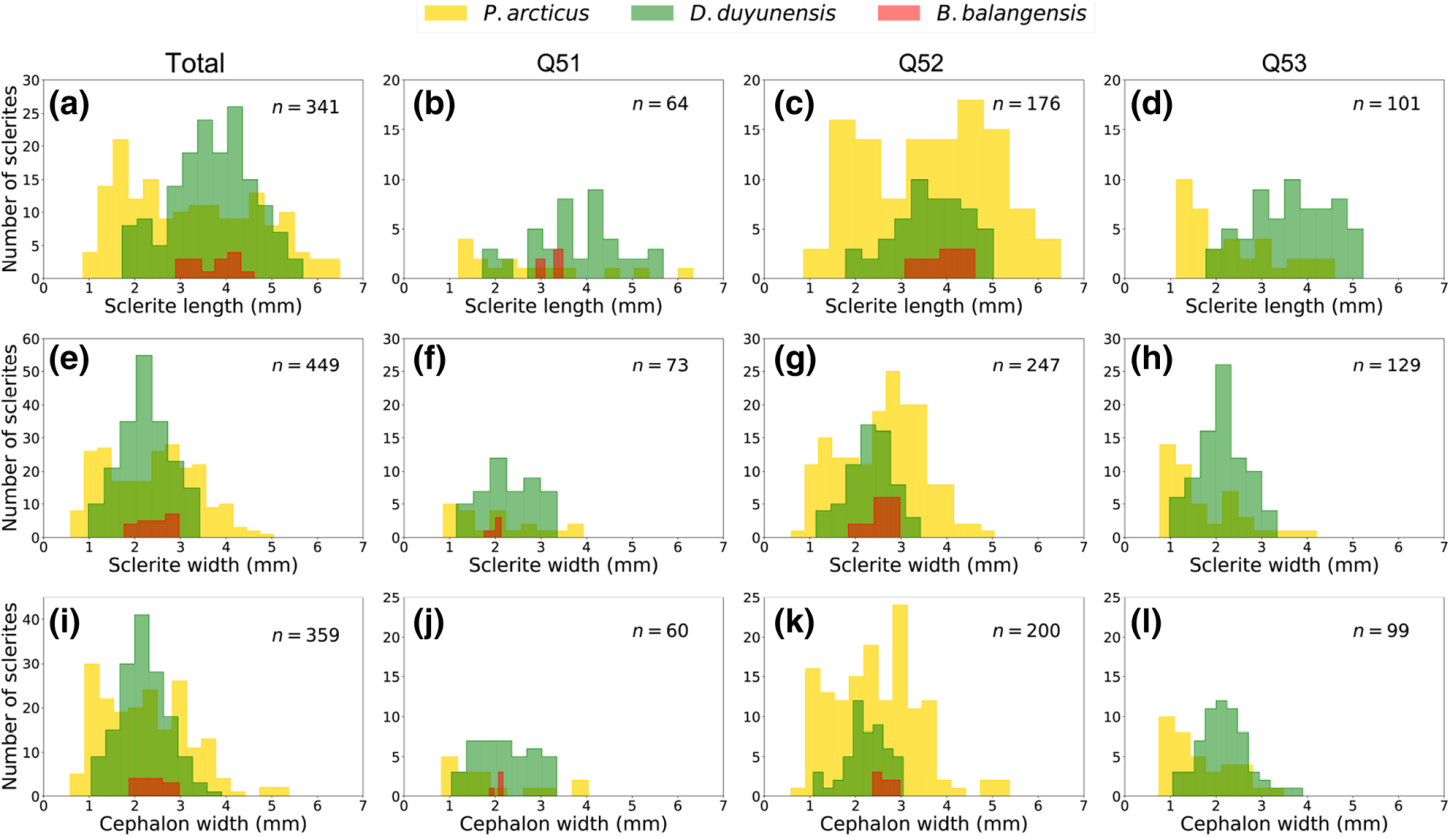
Size distribution of complete sclerites for three measures. **(a–d)** Sclerite length; **(e, f)** sclerite width; and **(i–l)** cephalic width in lower row. Analysis for each level: all individuals in first column, followed by Q51, Q52 and Q53. Plotted using Matplotlib library^65^.

In order to obtain the average size of each individual within the sample we measured three linear measurements: (i) maximum length; (ii) maximum width and (iii) maximum cephalic width. Although, *Protoryctocephalus arcticus* and *Duyunaspis duyunensis* were nearly equally abundant (359 and 336 individuals respectively), the size distribution of these species shows clear differences. *D. duyunensis* had a normal distribution while *P. arcticus* shows a bimodal distribution, most noticeably in specimens between 0.5 and 1.5 mm in width and specimens between 2.5 and 3.5 mm in width (Fig. 4). This bimodal distribution in *P. arcticus* may be explained by differences between the sample sites within the ‘Tsinghsutung’ Formation, however this seems to be well supported throughout Q52 and Q53, regardless of the type of measurement or the quantity of *D. duyunensis* (Fig. 5).

The ontogenetic series of these trilobites have not been studied yet but some of their developmental characters are noted. Both species show heminanamorphic development^29^ in which an anamorphic phase of development (i.e. sequential appearance of additional segments) is followed by an epimorphic phase (i.e. sequential moults retain a constant number of segments). This is used to separate the meraspid and holaspid stages. Axial length in the meraspid stage ranges from 0.86 to 5.68 mm in *P. arcticus* and from 1.72 to 5.18 mm in *D. duyunensis*; and maximum width from 0.59 to 3.30 mm in *P. arcticus* and from 0.98 to 3.09 mm in *D. duyunensis*.

### Dorso-ventral attitude and orientation

**Figure 6:**
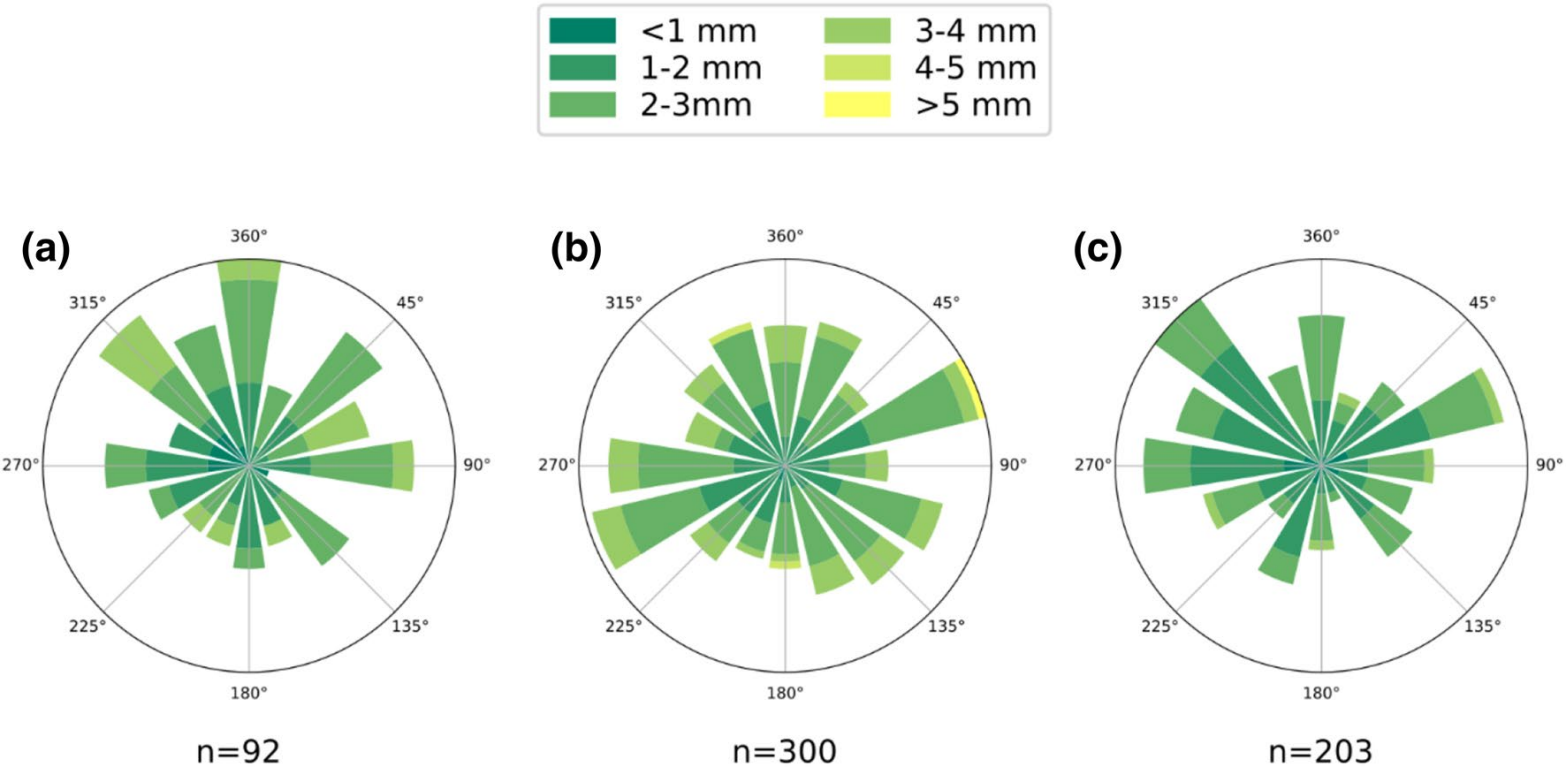
Orientation of complete sclerites for each level: **(a)** Q51, **(b)** Q52 and **(c)** Q53. Green color scales represent the sclerite length. Plotted using Windrose graphic tool^67^.

Dorso-ventral attitude and orientation are important features that can be employed to distinguish between trilobites that were accumulated biologically versus mechanically. The dorsal up attitude was dominant in two of the three beds (Q51 and Q53) and half of the samples in the case of Q52 are also dorsal up (Fig. 2). Given the size difference of the articulated specimens, samples displaying a width between 0.59 and 5.04 mm were divided into six different categories. No preferred orientation in any of the categories has been found in any of the three beds (Fig. 6). Although the sample from the Q52 bed shows a slightly denser orientation of ENE-WSW, the small K value (von Mises distribution) indicates a uniform distribution of the sample directions (0.01563, 0.03699, 0.08031).

### Moulting configurations and moulting behaviours

**Figure 7:**
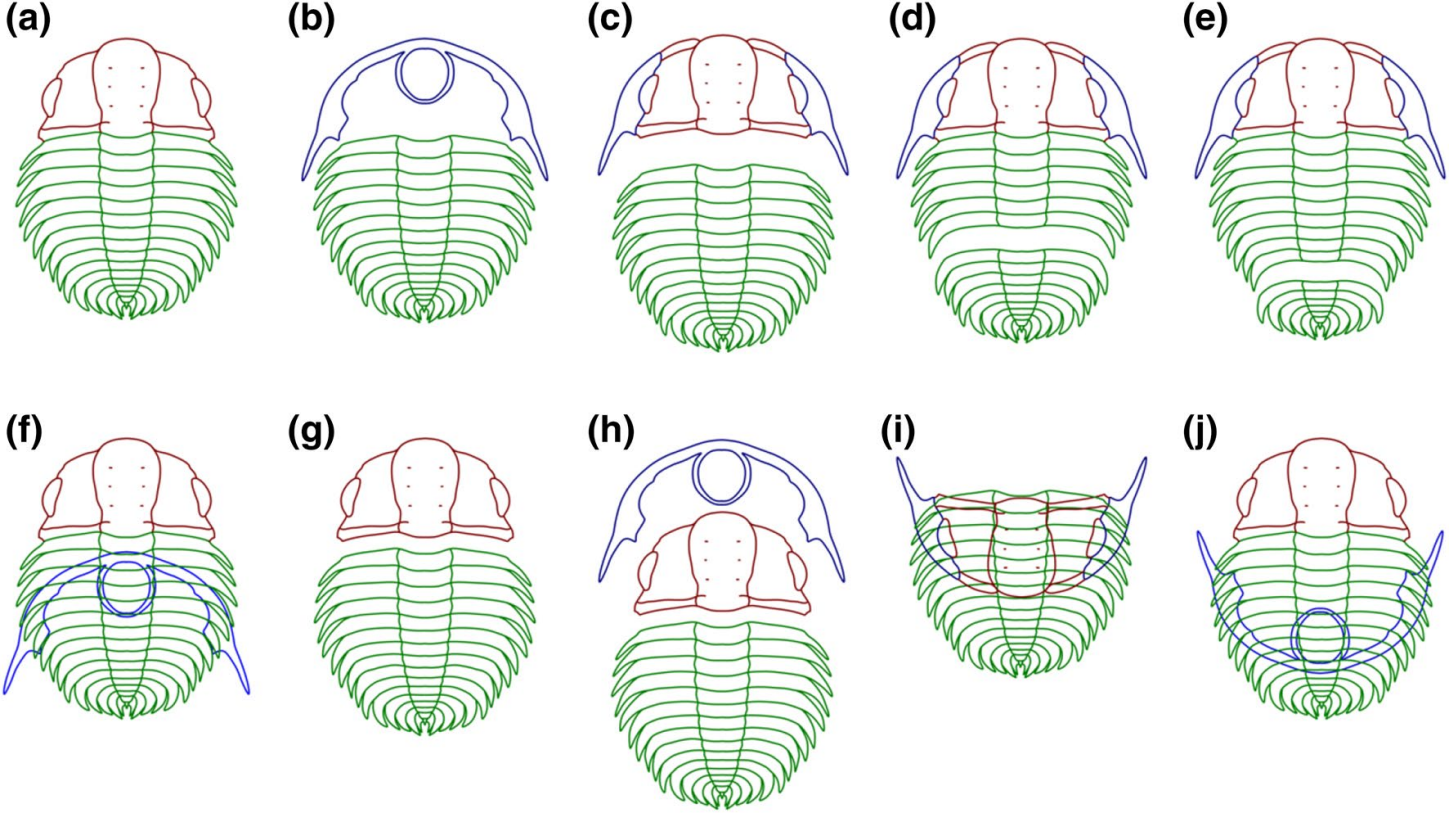
Moulting configurations found in this study. **(a)** Axial shield (n = 396); **(b)** shield with missing cranidium (a variant of Henningsmoen’s configuration) (n = 12); **(c)** cephalon disarticulated (n = 1); **(d)** disarticulation along the thorax (n = 3); **(e)** cephalotrunk with displaced pygidium (n = 2); **(f)** Maksimova’s configuration (n = 2); **(g)** facial and cephalotrunk sutures opened (with librigenae missing) (n = 18); **(h)** Henningsmoen’s configuration (n = 1); **(i)** Salter’s configuration (n = 4); **(j)** Somersault’s configuration (n = 2).

Our samples show every kind of moulting behaviour previously documented in the literature with a variety of moulting configurations. The majority of *P. arcticus* and *D. duyunensis* moults from the ‘Tsinghsutung’ Formation were found as axial shields (*c*. 46%, n = 396) (Fig. 7a). We found *c.* 3% of moults with Maksimova’s configuration (Fig. 7f), which is characterized by displaying a complete exoskeleton with the ventral cephalic unit displaced. These configurations agree with the ecdysial behaviour “facial sutures opened” (Fig. 3Sa–e). The assemblage also shows specimens with displaced cephalons or cranidium from the thorax forming an exuvial gape (Fig. 7c, g), which corresponds with the cephalon or cranidium removed behaviour (*c.* 2%, n = 19) (Fig. 3Sd–g, j). Four moults (*c.* 0.5%) had one or various dislocations along the thorax (Fig. 7d) and a large number of specimens were isolated thoracic segments (*c.* 19%, n = 159). This corresponds to the behaviour of thoracic dislocation (*c.* 0.3%, n = 3) (Fig. 3i). Only *c.* 14% (n = 117) of the assemblage are complete moults, although a few moults were nearly complete with only a missing pygidium (Fig. 7e), i.e. a pygidium—displaced behaviour (*c.* 0.2%, n = 2) (Fig. 3Sh). Shields missing the cranidium represented *c.* 1.4% (n = 12) of the moults (Fig. 7b) and there were also a few occurrences of Henningsmoen’s (Fig. 7h), Somersault’s (Fig. 7j) and Salter’s Configurations (Fig. 7i) (Less than 1%, n = 1, n = 2, n = 4). Approximately 9% (n = 79) of specimens were a combination of two moulting configurations (e.g. facial sutures opened, thoracic or pygidium dislocation), having disarticulated librigenae without the pygidium or the trunk. Additionally, isolated cephalic heads (*c.* 0.1%, n = 1) and cranidia (*c.* 5%, n = 42) were also present (Fig. 3Sl).

## Discussion and conclusions

### Justification of biological clustering behaviour

Trilobite clusters from the ‘Tsinghsutung’ Formation contain up to 857 specimens and occur in densely packed aggregates. Clusters are multitaxa but species are clearly segregated. The size distribution shows a trend towards very small sizes in all taxa (size segregated); consequently, the individuals in the clusters mostly represent early and late meraspid and early holaspid stages in the case of the oryctocephalids, and early holaspid stages in *B. balangensis*, although the latter is a small trilobite^28^. However, the abundance of each taxon shows a clear bias. The percentage of the two oryctocephalids is never equal, showing antagonist behaviour, and *B. balangensis* constitutes only a negligible percentage in all samples. Hydrodynamic sorting or current-aligned specimens would indicate at least local transportation^30^. The narrow size range of specimens preserved in all our samples from the three beds suggests that size-sorting, inferred from current transportation, is a possibility^31^. However, the absence of a preferred orientation within any sample suggests either the current velocity was not strong enough to re-orientate bioclasts, or there was a lack of consistency in current direction. Overall, the considerable size-sorting, the absence of current-induced sedimentary structures within each bed, and the presence of non-disrupted exuviae is suggestive of a low current velocity or even an absence of currents. On the other hand, the preservation of most of the trilobite bioclasts in a dorsal-up attitude may indicate that they were either (i) subject to unidirectional currents and adopted a hydrodynamically stable disposition; or (ii) were rapidly buried, since bioturbation can flip the exuviae. The dorsal-up attitude was the life attitude, but also resulted from normal exuviation in these trilobites. A number of studies on modern arthropods have demonstrated that disarticulation occurs within hours to weeks after death or ecdysis^25,30,32–36^. Therefore, given all this evidence, mechanical aggregation can be dismissed since there was no strong preferential currents and the bioturbation index is very low. Thus, it seems reasonable to state that our sample indicates behavioural congregation and that the specimens were buried rapidly after the ecdysis without any disturbance of the exuviae.

Trilobite clusters are commonly documented from the fossil record (e.g.^16,24,37–40^). Speyer and Brett^37^ suggested three possible scenarios to explain such behavioural congregation: (i) stress-related aggregation behaviour (e.g. storm disturbances); (ii) non-reproductive gregarious behaviour (e.g. moulting); and, (iii) reproductive clustering behaviour. The size sorting of the assemblage (i.e. meraspid stages *c.* 89%, n = 401) does not fit the profile of stress-related aggregation behaviour. If trilobites of all sizes were looking for shelter, as a result of stress, we should find a wide size range in the assemblage. Additionally, the taxonomic composition is very low, which is not indicative of a stress-related aggregation behaviour. Clusters or assemblages with high taxonomic diversification are related to faster and instantaneous burial such as obruption events^41,42^. On the other hand, most of the specimens represent early ontogenetic stages (mainly meraspid). Therefore, it seems very unlikely that these clusters represent reproductive clustering behaviour. The small size of specimens and the high number of moults is therefore suggestive that these clusters represent protection during moulting, reducing the risk of predation by moulting in large numbers in a quiet environment away from strong currents.

Ecdysis (i.e. moulting) is a critical stage for arthropods that determines its growth in the early stages and, in adults, allows reproduction as the soft body of the females allows for copulation and/or produces offspring^10,17,37^. Many extant groups of arthropods (e.g. crustacean, insects) undertake coordinated migrations^17,43^ and also possess a synchronized moulting cycle (a cycle also observed in trilobites (sensu^12,16,17,44^). The migration is triggered by the need for protection during this period of weakness (e.g. moulting), or alternatively increases the ability to find mates, as migrations bring many individuals together in one restricted geographic area. Trenchard et al*.*^45^ suggested that other trilobite genera such as oryctocephalids may have exhibited such behaviour because the deep-water areas are close to the exaerobic zones where the number of predators is reduced^39^. Clusters of juveniles or larvae are also common among arthropods and have been interpreted as a nursery^12,16,17^. Maintaining this gregarious behaviour could also carry other advantages for early larvae and juveniles, such as maternal food consumption (trophic egg consumption and/or matriphagy) and cooperative prey capture as observed in modern spiders^11,46,47^. Although our sample bears a high percentage of larvae, it is difficult to support such a hypothesis at this time, so further evidence is necessary to address this. Given the morphological features known in trilobites, they were unlikely to be aggressive with conspecifics. Although spines are present in their anatomy, these were likely for protection from larger predators and not from their own species^7^. Thus, they were apparently able to establish a gregarious behaviour and not suffer injuries among them. On the contrary, if they were aggressive, the moult synchrony would have helped to maintain the social coexistence given the inactivity before and during ecdysis reported in actual arthropods^5,11,12^.

### Moulting behaviour

The order Corynexochida was previously known to have four moulting behaviours: (i) Facial sutures opened; (ii) Rostral plate removed; (iii) Cephalon removed, and (iv) Cranidium removed (see^2^ for more details). The majority of moulting behaviours found here seem to be associated with a break between the cephalon and thorax along with a rupture of the facial suture produced by a downward dorsal flexure, which seems to be the trend within this group^2,48^. Nonetheless, we have found several moulting configurations which confirm that the rest of the moulting behaviours described by Daley and Drage^2^ and recently by Drage^6^ (but not figured) are also present in this trilobite order, suggesting that these behaviours were common across many trilobite orders. The available data shows that the most common moulting behaviour is the opening of the facial sutures, and the rest of the recognized behaviours are present in lower proportions. Our findings agree with the results reported by Daley and Drage^2^ and Drage^6^. However, given the high number of other behaviours reported in trilobites it seems unlikely that a relationship between configuration and behaviour exists. The sedimentological setting of the ‘Tsinghsutung’ Formation suggests a depositional environment with very little current action, where the trilobites were rapidly buried but not in a turbulent manner. Consequently, the biological meaning of the moulting configurations is uncertain. Further fieldwork to collect specimens within a sedimentological framework is necessary to assess the implications of such moulting behaviours for trilobite evolution.

This work shows one of the earliest synchronized moulting behaviours in the fossil record, and demonstrates that trilobites had complex social behaviours, providing a new insight into the ecological evolution of these early arthropods.

## Material and methods

### Geological setting and fossil assemblage

More than 10,000 specimens of *Protoryctocephalus arcticus*, *Burlingia balangensis* and *Duyanaspis duyonenesis* have been collected throughout the ‘Tsinghsutung’ Formation (123 to 250 m thick). The ‘Tsinghsutung’ Formation is within the transitional Jiangnan Slope Belt near the locality of Balang in the east of Guizhou Province, South China^49–51^. Our samples (857 specimens) were collected from three levels: 51, 52 and 53 m below the Kaili Formation (see Fig. 1S supplementary material and^52^). The lithology at this locality is unlike that of the Tsinghsutung or Wuxun formations studied previously in the transitional Jiangnan Slope Belt (see ^51,52^). This is why we have used inverted commas throughout the manuscript to indicate that the facies is similar to the Tsinghsutung Formation but with different lithological features. This formation in Balang is about 208 m thick and is mainly composed of grey to dark grey thick-bedded to massive limestone, grey thick-bedded dolomitic limestone, calcareous dolomite and grey thin- to thick-bedded dolomite and argillaceous dolomite. The samples in this study come from the *Protoryctocephalus arcticus* Zone. This zone occupies the middle-upper part of the ‘Tsinghsutung’ Formation and has a highly diverse trilobite assemblage: *Protoryctocephalus arcticus*, *Protoryctocephalus balangensis*, *Duyunaspis duyunensis*, *Redlichia noblis* (= *Redlichia guizhouensis*), *Burlingia balangensis*, *Panxinella angustilimbata, Dinesus panxinensis*, *Nangops danzhaicnsis*, *Balangcunaspis cransversu*, *Mufushania* cf. *nakingensis*, *Eosoptychoparia gaodongensis*, *Eosoptychoparia*sp. cf. *yunnanensis*, *Olenoides constrictus*, and *Olenoides*. cf. *hubeiensis*^53^.

All the samples were coated with ammonium chlorite prior to being photographed with a Nikon D300 camera. Length, width and angular measurements of the specimens were made using ImageJ^54^. Drawings were made using Adobe Illustrator CC2019.

### Taphonomy

Clusters can represent behavioural aggregation of individuals, but they can also be accumulated mechanically by means of currents. In this study we have followed several criteria (see below) for the identification a particular cluster type and, in turn, type of aggregation (behavioural or mechanical):

1. Type of preservation. The type of preservation tells us about the early diagenesis suffered by the trilobites, as dissolution or deep cementation can change morphological features^55^.
2. Percentage of articulation. Exoskeletons can be either fully articulated, which includes unbroken dorsal and ventral sutures, or partially articulated (as discussed above). Partially articulated individuals that do not represent obvious moult assemblages were also examined in terms of the proportion of various elements, i.e. cephala, axial shields, cephalothoraces and trunks. Those clusters with a high percentage of disarticulated sclerites likely represent a background time-averaging due to sediment starvation prior to burial.
3. Percentage of taxonomic composition. Clusters can be composed of either a single species (monotaxic) or two or more species (polytaxic). Those clusters with highly polytaxics and a high abundance of each taxon are suggestive of mechanical accumulation.
4. Size distribution. Individuals may be size-segregated (unimodal; but not necessarily implying a single cohort) or show a range of sizes (polymodal). A size segregation could be indicative of a stage-related biological behaviour or a size sorting due to environment conditions.
5. Orientation. Horizontal orientation is the facing direction of an individual within 360° on a horizontal bedding plane. Bioclast alignment in the azimuth orientation can provide an indication of current or wave transportation. This is especially accurate for the long axes of articulated exoskeletons^33^. Those unidirectional currents can re-orientate highly articulated specimens such that its sagittal axis is aligned with the current direction (anterior end pointing up-current), or such that its sagittal axis is perpendicular to the current direction, with the lateral margin facing into the current^56^.
6. Bioclast dorso-ventral attitude. Several experiments have demonstrated that complete, isolated cephala and cranidia adopt a hydrodynamically stable dorsal-up (convex-up) attitude when subjected to surface currents; in the absence of such currents, isolated cephala settle in an inverted (convex-down) attitude^25,56–59^. The dorso-ventral attitude of these sclerites can therefore offer biostratinomic insight^60–62^, with the caveats that any current-induced signal can be subsequently modified by surface scavenging (which results in predominantly convex-down sclerite attitudes; see^25,60^) and extensive bioturbation (which may result in random sclerite attitudes, including perpendicular or oblique to the sediment surface; see^25^).
7. Posture. Individuals may be prone (or outstretched) or display ventral flexure (including complete enrolment), dorsal flexure or torsion^63,64^. Posture, depending in the species, could indicate a rapid or slow burial.
8. Sedimentology. Sedimentary structures, lithology and microstratigraphic details (e.g., graded bedding within a microturbidite) may help elucidate the depositional setting and processes under which the cluster was preserved. The presence of certain structures could indicate direction of current flows, if present.
9. Fossils assemblage, paleoecology. The presence of other taxa may also be useful biostratinomic tools, especially biostratinomically sensitive, sessile multielement (e.g., echinoderms, *Wiwaxia*) and bivalved taxa (e.g., brachiopds)^68^.
10. Moults Vs Carcasses. In order to distinguish moults and carcasses we follow several criteria. The most important is to observe whether the specimens show suture lines with evidence of having been opened in one or several parts of the exoskeleton (e.g. both librigena)^2,48,60,63^. Other criteria, such as the lack of predation or scavenging evidences and lack of internal carcass features (i.e. guts) are also important criteria to distinguish moults and carcasses^2,13,25,37,39,40^.

### Statistical analysis

The data (measures, angles and morphology) were obtained and analysed using the ImageJ software (see supplementary material, dataset 1) and the resulting plots were made using Python 2D plotting library Matplotlib^65^. All quantitative data (percentages) were obtained using Python software. The K value of the Von Mises distribution was obtained using the open access Past software that uses the Rayleigh's test for directional data according to Davis^66^.

## Supplementary information

Supplementary Information.

Supplementary Figure 1.

Supplementary Figure 2.

Supplementary Figure 3.

Supplementary Dataset.

## References

[1] Owen AW (1985). Trilobite abnormalities. Earth Environ. Sci. Trans. R. Soc. Edinb..

[2] Daley AC, Drage HB (2016). The fossil record of ecdysis, and trends in the moulting behaviour of trilobites. Arthropod Struct. Dev..

[3] Clarkson EN (1969). On the schizochroal eyes of three species of Reedops (Trilobita: Phacopidae) from the Lower Devonian of Bohemia. Earth Environ. Sci. Trans. R. Soc. Edinb..

[4] Henningsmoen G (1975). Moulting in trilobites. Fossils Strata..

[5] Howe NR (1981). Partial molting synchrony in the giant Malaysian prawn, Macrobrachium rosenbergii: A chemical communication hypothesis. J. Chem. Ecol..

[6] Drage, H. B. Quantifying intra-and interspecific variability in trilobite moulting behaviour across the Palaeozoic. *Paleontol. Electron. 22*(2) (2019)**.**

[7] Pates S, Bicknell RD (2019). Elongated thoracic spines as potential predatory deterrents in olenelline trilobites from the lower Cambrian of Nevada. Palaeogeogr. Palaeoclimatol. Palaeoecol..

[8] Webster SG (1982). Seasonal anecdysis and moulting synchrony in field populations of Palaemon elegans (Rathke). Estuar. Coast. Shelf Sci..

[9] Leinaas HP (1983). Synchronized moulting controlled by communication in group-living Collembola. Science.

[10] Stone RP (1999). Mass molting of tanner crabs Chionoecetes bairdi in a Southeast Alaska-Estuary. Alaska Fish. Res. Bull..

[11] Kim KW (2001). Social facilitation of synchronized molting behavior in the spider Amaurobius ferox (Araneae, Amaurobiidae). J. Insect Behav..

[12] Haug JT, Caron JB, Haug C (2013). Demecology in the Cambrian: Synchronized molting in arthropods from the Burgess Shale. BMC Biol..

[13] Braddy SJ (2001). Eurypterid palaeoecology: Palaeobiological, ichnological and comparative evidence for a ‘mass–moult–mate’ hypothesis. Palaeogeogr. Palaeoclimatol. Palaeoecol..

[14] Karim T, Westrop SR (2002). Taphonomy and paleoecology of Ordovician trilobite clusters, Bromide Formation, south-central Oklahoma. Palaios.

[15] Vrazo MB, Braddy SJ (2011). Testing the ‘mass-moult-mate’hypothesis of eurypterid palaeoecology. Palaeogeogr. Palaeoclimatol. Palaeoecol..

[16] Paterson JR, Jago JB, Brock GA, Gehling JG (2007). Taphonomy and palaeoecology of the emuellid trilobite Balcoracania dailyi (early Cambrian, South Australia). Palaeogeogr. Palaeoclimatol. Palaeoecol..

[17] Błażejowski B, Brett CE, Kin A, Radwański A, Gruszczyński M (2016). Ancient animal migration: A case study of eyeless, dimorphic Devonian trilobites from Poland. Palaeontology.

[18] Vannier J, Vidal M, Marchant R, El Hariri K, Kouraiss K, Pittet B, Martin E (2019). Collective behaviour in 480-million-year-old trilobite arthropods from Morocco. Sci. Rep..

[19] Pocock KJ (1970). The Emuellidae, a new family of trilobites from the Lower Cambrian of South Australia. Palaeontology.

[20] Esker GC (1964). New species of trilobites from the Bromide Formation (Pooleville Member) of Oklahoma. Oklahoma Geology Notes..

[21] Thoral, M. *Contribution à l’étude paléontologique de l’Ordovicien inférieur de la Montagne Noire et révision sommaire de la faune cambrienne de la Montagne Noire*. (Imprimerie de la Charité, Montpellier, 1935).

[22] Passano, L. M. Molting and its control. In *Metabolism and Growth* (1960).

[23] Webster M, Gaines RR, Hughes NC (2008). Microstratigraphy, trilobite biostratinomy, and depositional environment of the “lower Cambrian” Ruin Wash Lagerstätte, Pioche Formation, Nevada. Palaeogeogr. Palaeoclimatol. Palaeoecol..

[24] Esteve J, Zamora S (2014). Enrolled agnostids from Cambrian of Spain provide new insights about the mode of life in these forms. Bull. Geosci..

[25] Speyer SE (1987). Comparative taphonomy and palaeoecology of trilobite lagerstätten. Alcheringa.

[26] Geyer G, Peel JS (2011). The Henson Gletscher Formation, North Greenland, and its bearing on the global Cambrian Series 2–Series 3 boundary. Bull. Geosci..

[27] Zhou, T. M., Liu, Y. R., Meng, X. S & Sun, Z. H. Palaeontological atlas of central and southern China. In *Early Palaeonzoic*, vol. 1 (eds. Hubei Institute of Geological Sciences, Geological Bureau of Henan Province, Geological Bureau of Hubei Province, Geological Bureau of Hunan Province, Geological Bureau of Guangdong Province & Geological Bureau of Guangxi Province) 104–266 (Geological Publishing House, Beijing, 1977).

[28] Yuan JL, Esteve J (2015). The earliest species of *Burlingia* Walcott, 1908 (Trilobita) from South China: Biostratigraphical and palaeogeographical significance. Geol. Mag..

[29] Hughes NC, Minelli A, Fusco G (2006). The ontogeny of trilobite segmentation: A comparative approach. Paleobiology..

[30] Brett CE, Baird GC (1993). Taphonomic approaches to temporal resolution in stratigraphy: Examples from Paleozoic marine mudrocks. Short Courses Paleontol..

[31] Brandt DS (1989). Taphonomic grades as a classification for fossiliferous assemblages and implications for paleoecology. Palaios.

[32] Schäfer W, Oertel I (1972). Ecology and Palaeoecology of Marine Environments.

[33] Brett CE, Baird GC (1986). Comparative taphonomy: A key to paleoenvironmental interpretation based on fossil preservation. Palaios.

[34] Plotnick, R. E. Taphonomy of a modern shrimp: Implications for the arthropod fossil record. *Palaios.* 286–293 (1986).

[35] Plotnick RE, Baumiller T, Wetmore KL (1988). Fossilization potential of the mud crab, Panopeus (Brachyura: Xanthidae) and temporal variability in crustacean taphonomy. Palaeogeogr. Palaeoclimatol. Palaeoecol..

[36] Babcock LE, Chang W (1997). Comparative taphonomy of two nonmineralized arthropods: Naraoia (Nektaspida; Early Cambrian, Chengjiang Biota, China) and Limulus (Xiphosurida; Holocene, Atlantic Ocean). Collect. Res..

[37] Speyer SE, Brett CE (1985). Clustered trilobite assemblages in the Middle Devonian Hamilton group. Lethaia..

[38] Paterson JR, Hughes NC, Chatterton BD, Rábano I, Gozalo R, García-Bellido D (2008). Trilobite clusters: What do they tell us? A preliminary investigation. Adv. Trilobite Res..

[39] Gaines RR, Droser ML (2003). Paleoecology of the familiar trilobite *Elrathia kingii*: An early exaerobic zone inhabitant. Geology.

[40] Gutiérrez-Marco JC, Sá AA, García-Bellido DC, Rábano I, Valério M (2009). Giant trilobites and trilobite clusters from the Ordovician of Portugal. Geology.

[41] Esteve J, Hughes NC, Zamora S (2011). Purujosa trilobite assemblage and the evolution of trilobite enrollment. Geology.

[42] Brett CE, Zambito JJ, Schindler E, Becker RT (2012). Diagenetically-enhanced trilobite obrution deposits in concretionary limestones: The paradox of “rhythmic events beds”. Palaeogeogr. Palaeoclimatol. Palaeoecol..

[43] Hoare, B. *Animal Migration: Remarkable Journeys in the Wild*. (University of California Press, 2009).

[44] Chatterton, B. D. E. & Fortey, R. A. Linear clusters of articulated trilobites from Lower Ordovician (Arenig) strata at Bini Tinzoulin, North Zagora, Southern Morocco. *Adv. Trilobite Res. *(Cuadernos del Museo Geominero) **9**, 73–77 (2008).

[45] Trenchard H, Brett CE, Perc M (2017). Trilobite ‘pelotons’: Possible hydrodynamic drag effects between leading and following trilobites in trilobite queues. Palaeontology.

[46] Kim KW, Horel A (1998). Matriphagy in the spider *Amaurobius ferox* (Araneidae, Amaurobiidae): an example of mother-offspring interactions. Ethology.

[47] Kim KW, Roland C (2000). Trophic egg laying in the spider, *Amaurobius ferox*: mother–offspring interactions and functional value. Behav. Proc..

[48] Drage HB, Holmes JD, García-Bellido DC, Daley AC (2018). An exceptional record of Cambrian trilobite moulting behaviour preserved in the Emu Bay Shale, South Australia. Lethaia.

[49] Zhao, Y. L. *et al.* Balang section, Guizhou, China: Stratotype section for the Taijiangian Stage and candidate for GSSP of an unnamed Cambrian Series. *Camb. Syst. China Korea Guide Field Excursions* 62–83 (2005).

[50] Zhao YL (2005). Kaili Biota: A taphonomic window on diversification of metazoans from the basal Middle Cambrian: Guizhou, China. Acta Geol. Sin.-English Ed..

[51] Yang XL, Zhao YL, Peng J, Yang YN, Yang KD (2010). Discovery of Oryctocephalid trilobites from the Tsinghsutung Formation (Duyunian Stage, Qiandongian Series, Cambrian), Jianhe County, Guizhou Province. Geol. J. China Univ..

[52] Yuan JL, Esteve J, Ng TW (2014). Articulation, interlocking devices and enrolment in *Monkaspis* daulis (W alcott, 1905) from the Guzhangian, middle Cambrian of North China. Lethaia..

[53] Zhao YL, Yuan JL, Esteve J, Peng J (2017). The oryctocephalid trilobite zonation across the Cambrian Series 2-Series 3 boundary at Balang, South China: A reappraisal. Lethaia..

[54] Abràmoff MD, Magalhães PJ, Ram SJ (2004). Image processing with ImageJ. Biophoton. Int..

[55] Esteve J, Zhao YL, Maté-González MA, Gómez-Heras M, Peng J (2018). A new high-resolution 3-D quantitative method for analysing small morphological features: An example using a Cambrian trilobite. Sci. Rep..

[56] Lask, P. B. The hydrodynamic behavior of sclerites from the trilobite Flexicalymene meeki. *Palaios*, 219–225 (1993).

[57] Hesselbo, S. P. The biostratinomy of *Dikelocephalus* sclerites: implications for the use of trilobite attitude data. *Palaios.* 605–608 (1987).

[58] Mikulic DG (1990). The arthropod fossil record: biologic and taphonomic controls on its composition. Short Courses Paleontol..

[59] Speyer, S. E. & Donovan, S. K. Trilobite taphonomy: A basis for comparative studies of arthropod preservation, functional anatomy and behaviour. *Processes Fossil.*, 194–219 (1991).

[60] Speyer, S. E. & Brett, C. E. Trilobite taphonomy and Middle Devonian taphofacies. *Palaios.,* 312–327 (1986).

[61] Schumacher, G. A. & Shrake, D. L. Paleoecology and comparative taphonomy of an *Isotelus* (Trilobita) fossil lagerstätten from the Waynesville Formation (Upper Ordovician, Cincinnatian Series) of southwestern Ohio. In *Paleontological Events: Stratigraphic, Ecological, and Evolutionary Implications. *131–161 (Columbia University Press, New York, 1997).

[62] Hickerson, W. J. Middle Devonian (Givetian) trilobite clusters from eastern Iowa and northwestern Illinois. In *Paleontological Events: Stratigraphic, Ecological, and Evolutionary Implications. *224–246 (Columbia University Press, New York, 1997).

[63] Hughes NC, Cooper DL (1999). Paleobiologic and taphonomic aspects of the “granulosa” trilobite cluster, Kope Formation (Upper Ordovician, Cincinnati region). J. Paleontol..

[64] Hunda, B. R., Hughes, N. C. & Flessa, K. W. Trilobite taphonomy and temporal resolution in the Mt. Orab shale bed (Upper Ordovician, Ohio, USA). *Palaios.* **21(1)**, 26–45 (2006).

[65] Hunter JD (2007). Matplotlib: A 2D graphics environment. Comput. Sci. Eng..

[66] Davis, J. C. Statistics and data analysis In *Geology* 289–291 (Wiley, New York, 1986).

[67] Roubeyrie L, Celles S (2018). Windrose: A Python Matplotlib, Numpy library to manage wind and pollution data, draw windrose. J Open Source Softw..

[68] Sun H-J, Zhao Y-L, Peng J, Yang Y-N (2014). New *Wiwaxia* material from the Tsinghsutung Formation (Cambrian Series 2) of Eastern Guizhou, China. Geol. Mag..

