# Supplementary material for: Synchronized moulting behaviour in trilobites from the Cambrian Series 2 of South China
Source: Sci Rep. 2020 Aug 24;10:14099. doi: 10.1038/s41598-020-70883-5 (PMC7445173; doi:10.1038/s41598-020-70883-5)
Supplement: Supplementary file 1 — Supplementary Information. [file 41598_2020_70883_MOESM1_ESM.docx]

**Synchronized moulting behaviour in trilobites from the Cambrian Series 2 of South China**

Alejandro Corrales-García^1^, Jorge Esteve^1,*^, Zhao Yuanlong^2^ & Yang Xinglian^2^

1 Department of Geosciences, Universidad de Los Andes, Bogotá D.C., 111711, Colombia

2 Resources and Environmental Engineering College, Guizhou University, Guiyang, 550025, China

^*^ Corresponding authors [*jv.esteve@uniandes.edu.co* and *yangxinglian2002@ 163.com*]

**Supplementary Information**


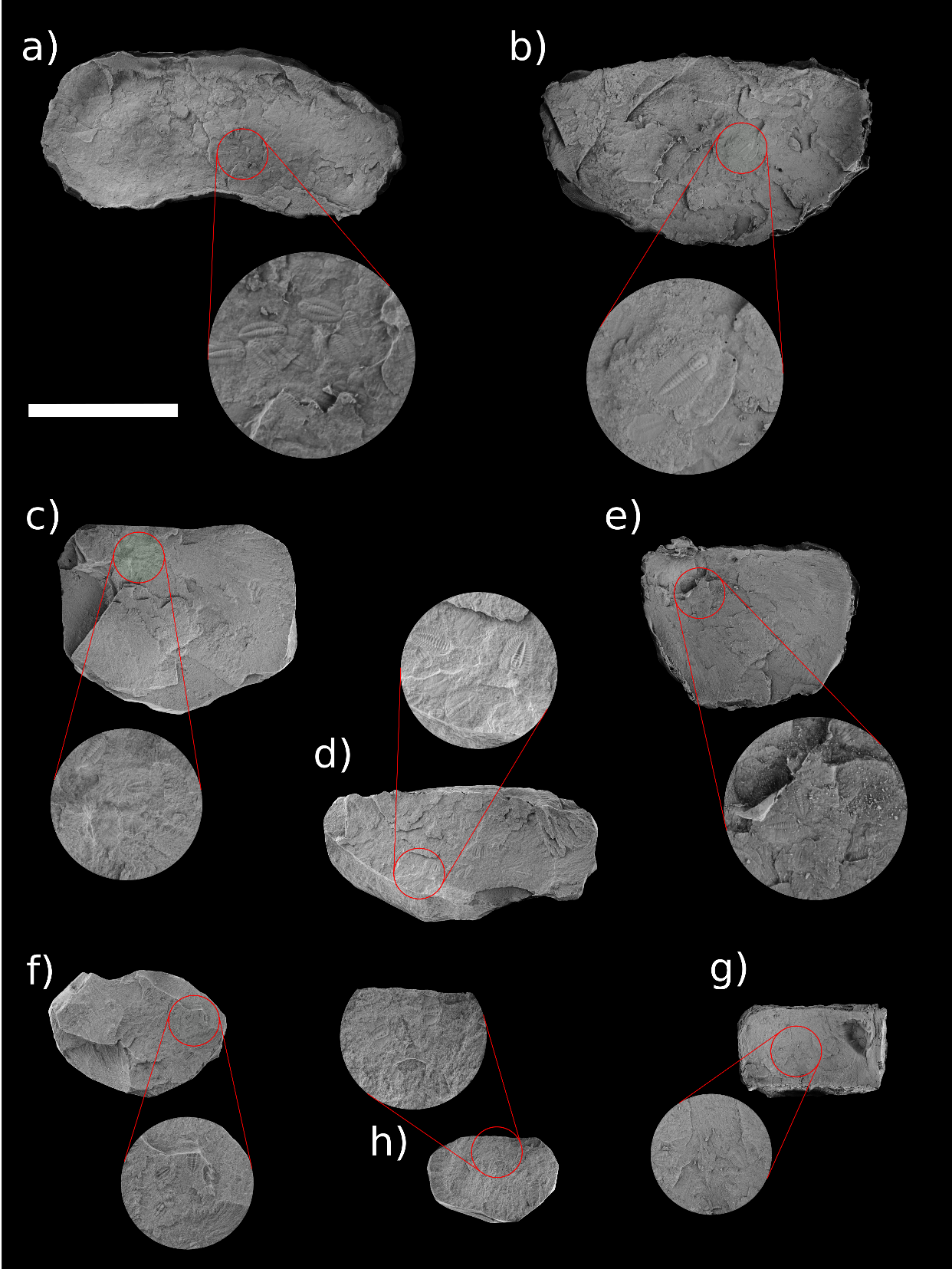


Figure 1S. Slabs of trilobite clusters. a) Q53-15; b) Q52-2391; c) Q51-1213; d) Q51-12188; e) Q51-1216; f) Q52-674; g) Q51-1207. Scale bar = 3 cm


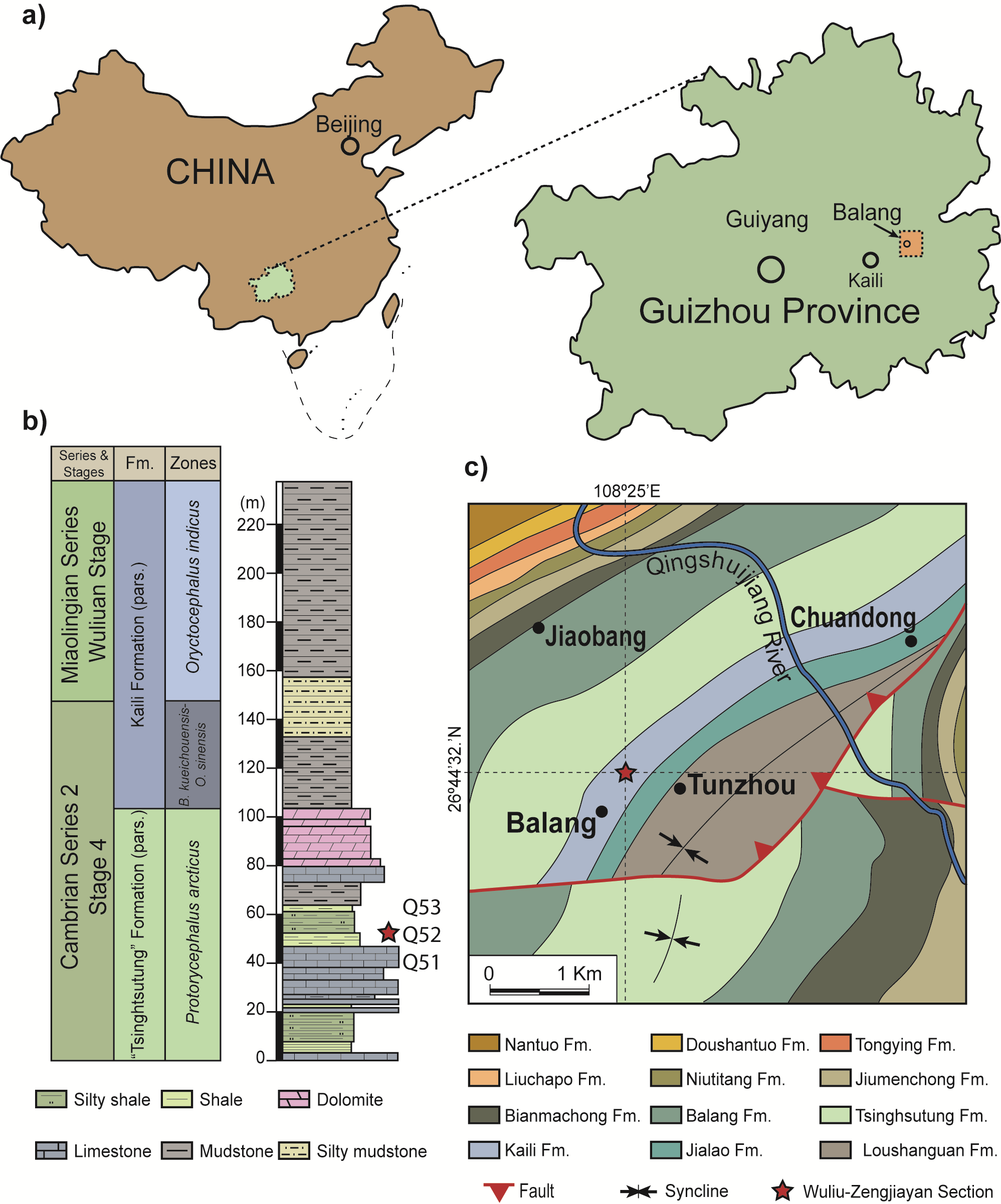


Figure 2S. a-c) Geological setting. A, Map of China showing Guizhou Province and the Balang locality; b) Stratigraphic column of the upper part of the ‘Tsinghsutung’ Formation and the lower part of the Kaili Formation of the Malipo section showing the location of the clusters; c) Geological map of the Balang area indicating the location where the cluster were collected.


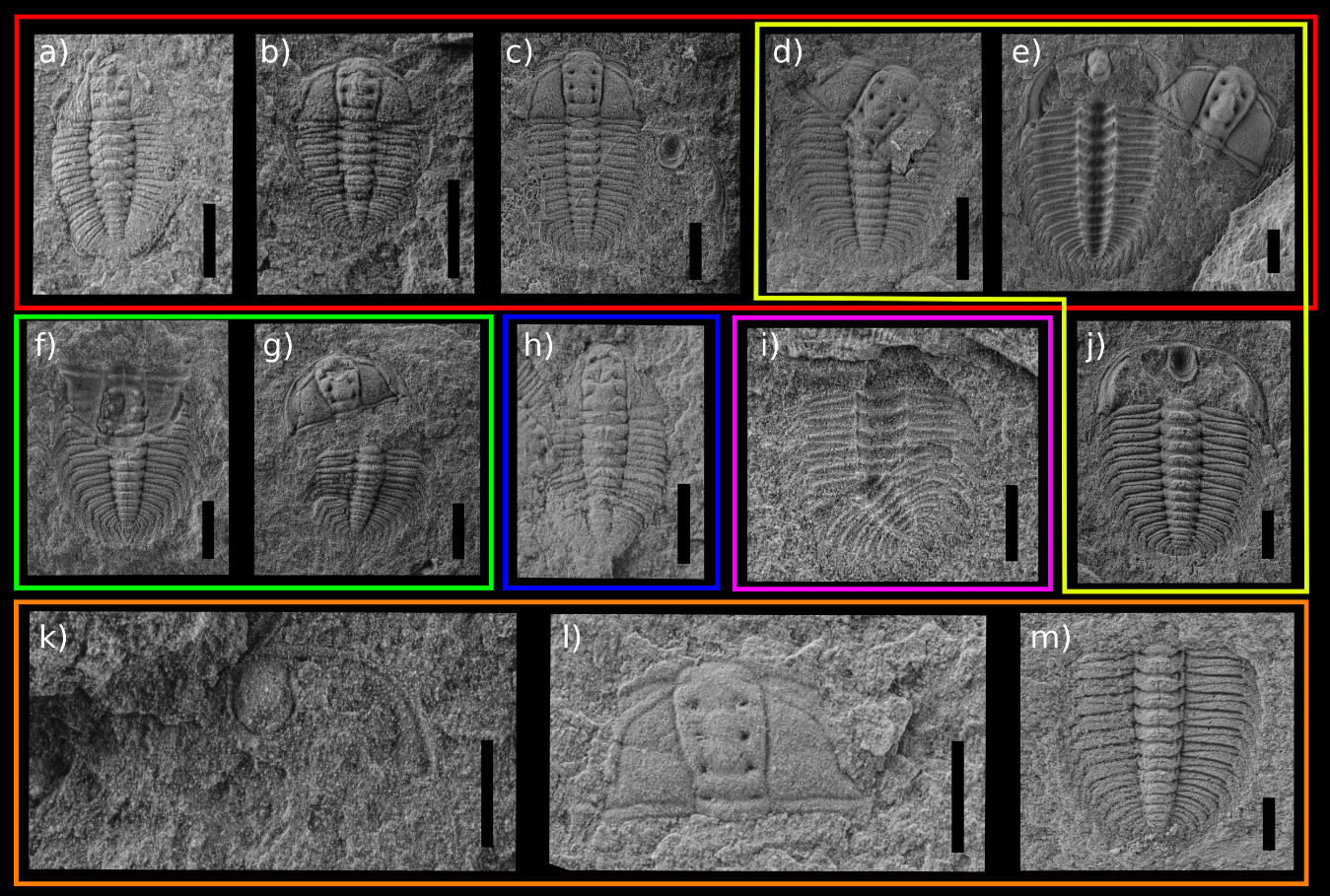


Figure 3S. Trilobite moulting configurations and isolated parts found in the ‘Tsinghsutung’ Formation: a) axial shield (Q51-1213); b) Somersault’s configuration (Q52-123); c) Maksimova’s configuration (DCS-7871); d) facial and cephalotrunk sutures opened (Q52-45); e) Henningsmoen’s configuration (DSC--8283) (Q52-1123); f) Salter’s configuration (); g) cephalon disarticulated (); h) cephalotrunk with displaced pygidium (Q51-1213); i) disarticulation along the thorax (Q52-891); j) shield with missing cranidium (variant of Henningsmoen’s configuration) (); k) lower cephalic unit (Q51-652); l) isolated cranidium (Q52-4675); m) isolated trunk part (Q52-2325). In colour moulting behaviours: red, facial sutures opened; yellow, cranidium removed; green, cephalon removed; blue, pygidium displaced; pink, thoracic dislocation; orange, isolated parts.
