# Supplementary figures and images for: Synchronized moulting behaviour in trilobites from the Cambrian Series 2 of South China
Source: Sci Rep. 2020 Aug 24;10:14099. doi: 10.1038/s41598-020-70883-5 (PMC7445173; doi:10.1038/s41598-020-70883-5)

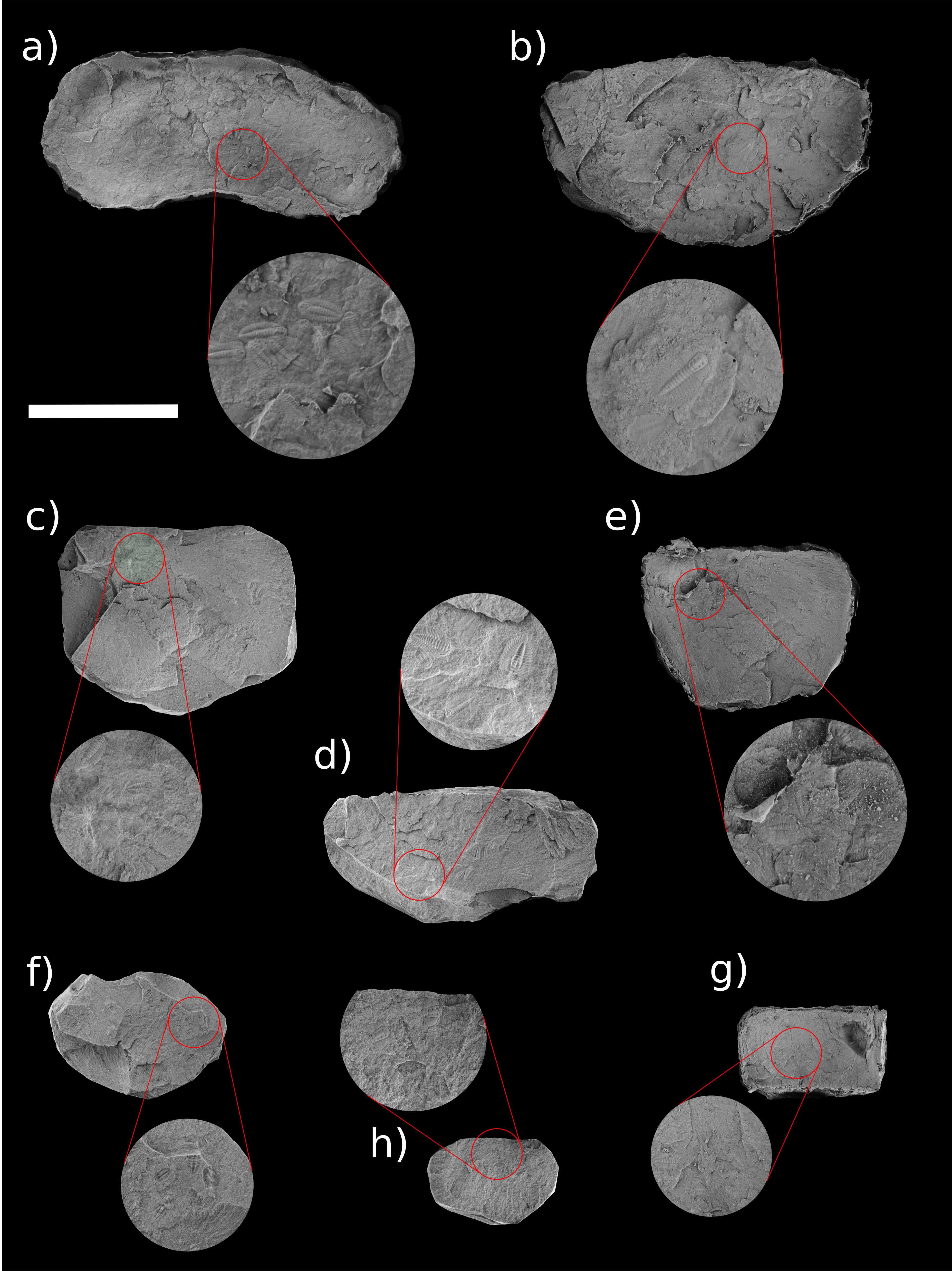

Supplement: Supplementary file 2 — Supplementary Figure 1. [file 41598_2020_70883_MOESM2_ESM.png]

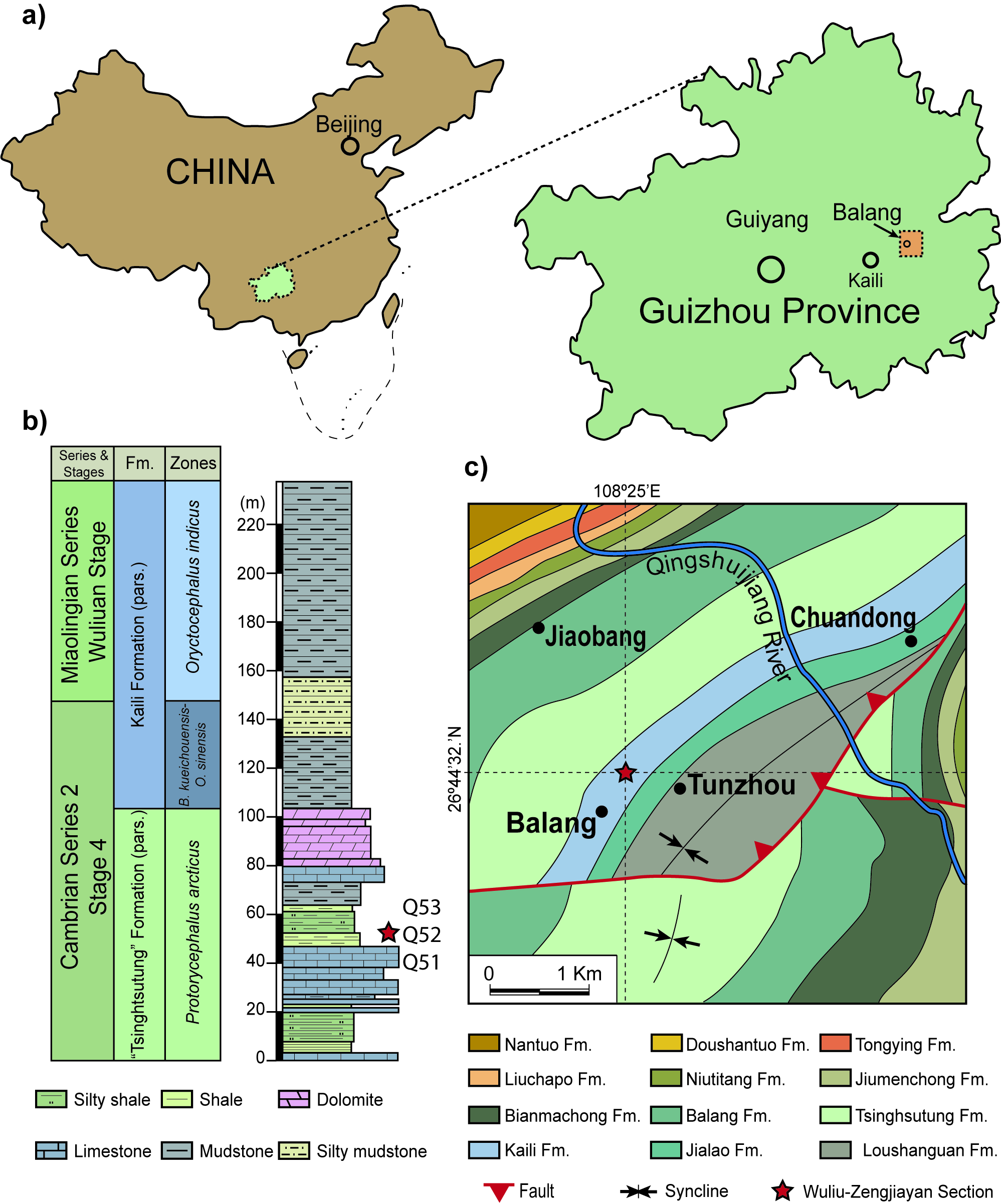

Supplement: Supplementary file 3 — Supplementary Figure 2. [file 41598_2020_70883_MOESM3_ESM.tif]

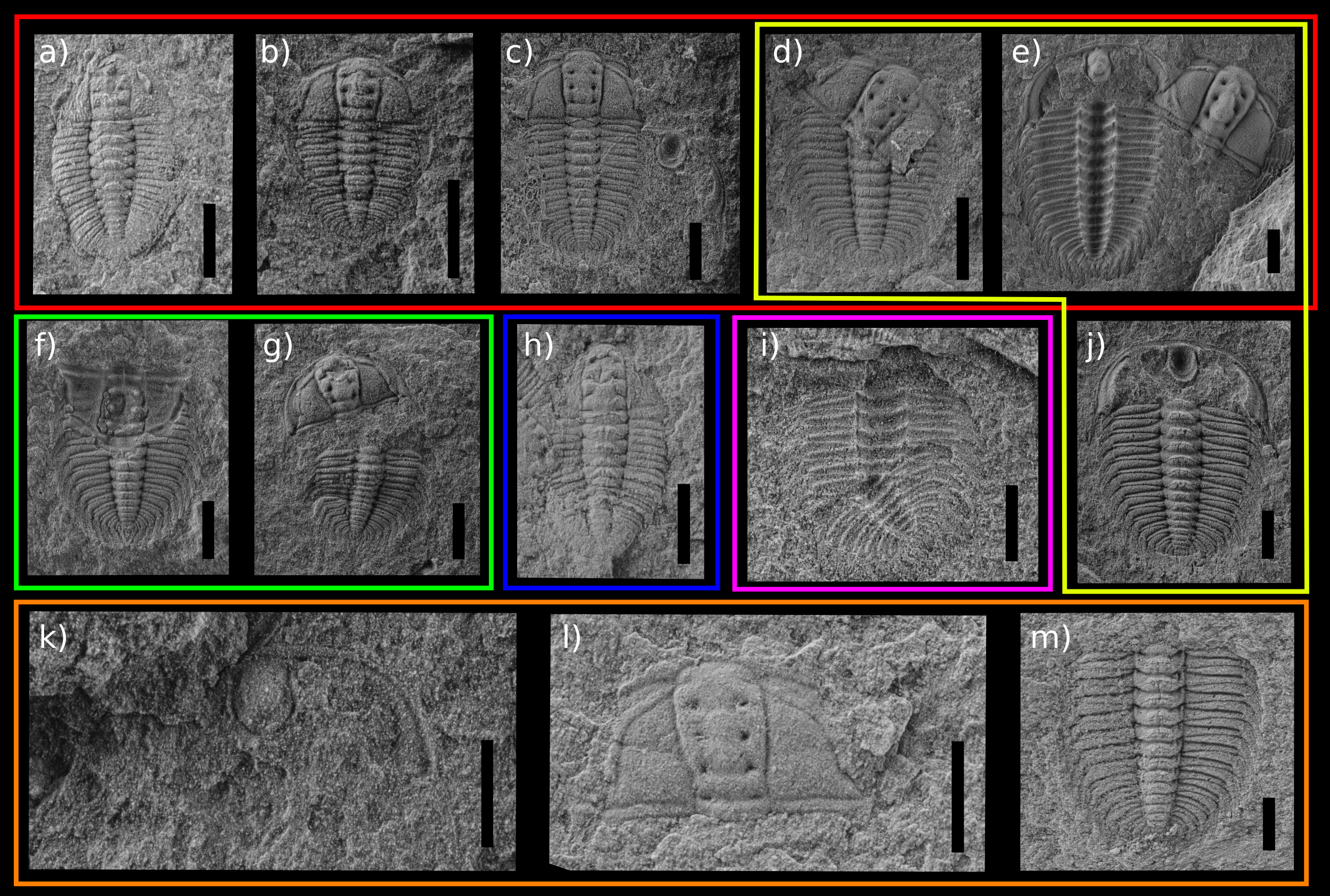

Supplement: Supplementary file 4 — Supplementary Figure 3. [file 41598_2020_70883_MOESM4_ESM.png]
